# Impact of Resistance Therapy on Motor Function in Children with Cerebral Palsy: A Systematic Review and Meta-Analysis

**DOI:** 10.3390/ijerph16224513

**Published:** 2019-11-15

**Authors:** Luisa Collado-Garrido, Paula Parás-Bravo, Pilar Calvo-Martín, Miguel Santibáñez-Margüello

**Affiliations:** 1 Rehabilitation Service, The Marqués de Valdecilla University Hospital, Valdecilla Avenue s/n. C.P.: 39008 Santander, Cantabria, Spain; luisacolladogarrido@yahoo.es (L.C.-G.); mpilar.calvo@scsalud.es (P.C.-M.); 2 Faculty of Nursing, University of Cantabria, Valdecilla Avenue s/n., 39008 Santander, Cantabria, Spain; miguel.santibanez@unican.es; 3 Research Nursing Group IDIVAL; Cardenal Herrera Oria Street s/n. C.P., 3901 Cantabria, Spain; 4 Global Health Research Group, University of Cantabria, 39008 Santander, Cantabria, Spain

**Keywords:** Cerebral palsy, resistance therapy, motor skills, child, meta-analysis

## Abstract

Cerebral palsy is one of the main causes of disability in childhood. Resistance therapy shows benefits in increasing strength and gait in these patients, but its impact on motor function is not yet clear. The objective was to analyze the impact of resistance therapy on the improvement in the motor function using a review and meta-analysis. A comprehensive literature research was conducted in Medline (PubMed), Institute for Scientific Information (ISI) Web of Knowledge, and Physiotherapy Evidence Database (PEDro) in relation to clinical trials in which resistance therapy was used and motor function was assessed. Twelve controlled clinical trials and three non-controlled clinical trials (only one intervention arm) studies were identified. In terms of pre–post difference, the overall intra-group effect was in favor of resistance therapy intervention: standardized mean difference (SMD) = 0.37, 95% confidence interval (CI) = 0.21 to 0.52, *p* < 0.001 (random-effects model), with moderate heterogeneity (*I^2^* = 59.82%). SMDs were also positive by restricting to each of the analyzed scales: SMD = 0.37, 1.33, 0.10, and 0.36 for Gross Motor Function Measure (GMFM), Lateral Step Up (LSU), Time Up and Go (TUG), and Mobility Questionnaire (MobQue) scales, respectively. Regarding the difference between groups, the results showed a high heterogeneity (*I^2^* < 99%), with the mean difference (MD) also favorable for the GMFM scale: MD = 1.73, 95% CI = 0.81 to 2.64, *p* < 0.001 (random-effects model). Our results support a positive impact of resistance therapy on motor function. Further studies should delve into the clinical relevance of these results.

## 1. Introduction

Cerebral palsy (CP) is currently the most common cause of motor disability in the pediatric population. In the last 40 years, the incidence of CP increased well above 2.0 per 1000 live births in developed countries [1].

Most children with CP show a significant weakness in spastic musculature compared with the least affected. In this sense, current evidence suggests that muscle weakness in CP may contribute to disability to a greater extent than spasticity itself [2].

In the past, it was feared that the negative effect of increased spasticity associated with resistance therapy interventions was greater than the positive effect associated with these therapies on muscle strength, gait, and motor function [3].

However, a systematic review published in 2002 suggested that resistance exercises could increase muscle strength without increasing spasticity [4], opening up the possibility that this increase in strength could be simultaneously associated with improvements in gait or motor function.

Several subsequent primary studies led firstly to the publication of a review in 2009 [5], in which results were found in favor of motor function based on four published primary studies evaluated. In 2014, in the Park et al. meta-analysis [6], the authors concluded that resistance therapy improves muscle strength and that this increase would also affect gait parameters, establishing an intervention protocol of 40–50 min three days per week. Regarding the motor function, the results were inconclusive, highlighting the need for more specific studies. The latest meta-analysis published according to our knowledge as a Cochrane review in 2017 [7] did not find a statistically significant increase in the specific dimension of the motor function determined using the GMFM scale (Gross Motor Function Measure) based on the seven primary articles found during their search until June 2016 [8,9,10,11,12,13,14].

These inconclusive results regarding motor function may be due both to heterogeneity in the study population, as not all reviews published to date focused on children [4,6,7], and to heterogeneity in resistance therapy interventions [6,15,16], as electrical stimulation, for example, was included within these interventions, when it is actually not resistance work, but involuntary muscle enhancement [17]. Finally, the heterogeneity in the quality of the methodologies applied in the primary studies [5,6,7] may also explain the differential results.

These inconclusive results support the need for a systematic review and meta-analysis to know the specific impact of resistance therapy on motor function, taking into account these sources of heterogeneity. A meta-analysis that synthesizes the quantitative measures found in relation to resistance therapy in children in a more homogeneous way, and that incorporates a subgroup analysis based on the quality of the identified studies would be very useful to establish optimal treatment protocols. 

Therefore, the objective of this study was to analyze, through a meta-analysis of published primary studies, the impact of resistance therapy on the parameters of the motor function in children with CP.

## 2. Materials and Methods

A bibliographic search was conducted to identify epidemiological studies carried out on school-aged CP patients (≤18 years), written in English or Spanish, in which resistance therapy was used and which reported at least one determination in motor function, both pre and post intervention, in order to assess intra-group change and those that reported differences between groups in this outcome measure. Different international bibliographic databases were consulted: Medline through PubMed, Institute for Scientific Information (ISI) Web of Knowledge, and Physiotherapy Evidence Database (PEDro). All relevant primary studies (published and under publication) until January 2018 were identified, using the following keywords: “strength training” OR “strengthening” OR “resistance exercise” AND “cerebral palsy”, using free text and without applying any limitation in the search strategy. We also performed a manual search within the bibliographic references of the retrieved studies. In total, 631 primary studies were found in the search in Medline, 1034 in ISI Web of Knowledge, and 130 in PEDro.

**Figure 1:**
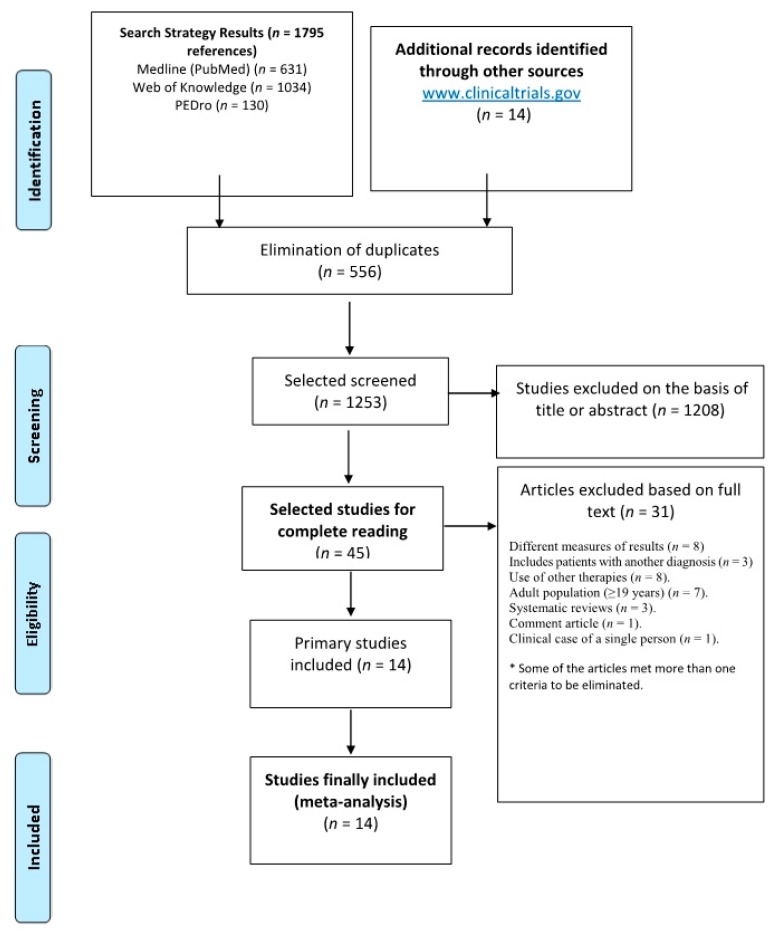
Flowchart used for the identification of clinical trials and studies with intervention based on resistance therapy and motor function measured as the outcome variable.

Studies using electrostimulation as resistance therapy and populations with diseases other than CP were excluded. Table 1 shows the inclusion and exclusion criteria applied to the references found, either by reading the abstracts or, when necessary, by reading the full text of the primary studies. Figure 1 shows the flowchart used to identify the primary studies to be included in the review, and it also reports the reasons for exclusion. For the identification of those ongoing studies, we searched the electronic database of clinical trial registries: Current Controlled Trials, National Health Service, The National Research Register, and Clinical Trials.

The assessment of the presence of the main types of biases and of the overall methodological quality in each primary study was carried out in a standardized manner using the tool known as the PEDro Scale [18,19,20]. 

Quality assessment followed the recommendations of Chalmers [21] and Santibañez [22] in order to minimize observer bias; each article was assigned an identification number, eliminating journal and author data. Each primary study was assessed independently by two reviewers (L.C.-G. and M.S.-M.). In cases of discrepancy in the evaluation, it was assessed whether the discrepancy affected the qualitative rate or the quantitative score, resolved by consensus.

### Data Analysis

The standardized mean difference (SMD), with its 95% confidence interval (95% CI), was chosen as a summary measure of the effect to allow us to combine data for the following motor function scales: Gross Motor Function Measure (GMFM), Lateral Step Up (LSU), Time Up and Go (TUG), and Mobility Questionnaire (MobQue) used in the meta-analysis. This strategy, which is consistent with the approach taken in other reviews [7,23], increases the pool of studies, thereby increasing the power to detect both intra-group and between-group differences in the motor functions.

In a second strategy, as a sensitivity analysis, the results were restricted to studies using the GMFM scale. In this second approach, the mean difference (MD) on the natural (non-standardized) scale was used.

To weight intervention effects, a random-effects model versus a fixed-effects model was chosen after studying the heterogeneity for each outcome. Statistical heterogeneity was assessed through Cochran’s Q-test and *I^2^* statistic, which describe the percentage of total variation across studies that is attributable to statistical heterogeneity rather than to chance. *I^2^* values of 25%, 50%, and 75% correspond to low, moderate, and high between-study statistical heterogeneity. A *p*-value <0.10 was set as the cut-off point for statistically significant heterogeneity in the chi-squared test for heterogeneity [24]. We used the DerSimonian and Laird random-effects model with inverse variance to generate SMDs and MDs [25]. 

Subgroup analyses were predefined, attending to the study design, duration of therapy, number of sessions, duration of each session, and type of intervention protocol, depending on the score in the analysis of methodological quality.

We sought evidence of publication bias using the funnel plot method and Egger’s regression asymmetry test [26,27]. In addition, Duval and Tweedie’s “trim and fill” approach was used to obtain the best estimation of the unbiased effect size [28]. 

The meta-analysis was written following the recommendations of the Preferred Reporting Items for Systematic Reviews and Meta-Analyses (PRISMA) statement [29]. All analyses were conducted using Comprehensive Meta-Analysis (CMA v2) [30]. 

## 3. Results

According to the selection criteria, 15 original articles were found; 12 were randomized controlled clinical trials [8,9,10,12,13,14,31,32,33,34,35,36], all of them with two parallel branches (arms), and three were non-controlled clinical trials (only one intervention arm) [37,38,39]. Table 2 presents the characteristics of these finally included studies. 

Regarding the assessment of the overall methodological quality in each primary study, two of the studies obtained an “excellent” score (7–8 points out of 10) [32,36], seven studies obtained a “good” score (between five and six points) [8,12,13,14,31,34,35], four studies obtained a “fair” score (between three and four points) [9,10,33,39], and two of them obtained a “poor” score (two points) [37,38] (see Table 3).

### 3.1. Intra-Group Pre–Post Difference in the Intervention Group

Fifteen studies [8,9,10,12,13,14,31,32,33,34,35,36,37,38,39] provided data related to a resistance therapy intervention that could be meta-analyzed for the evaluation of the pre–post intra-group difference in the motor function. These 15 studies used up to four different scales (GMFM, MobQue, LSU, and TUG). Twelve of these studies provided data on the GMFM scale, three of these studies provided data on the TUG and LSU scales, and two studies provided data on the MobQue scale.

**Figure 2:**
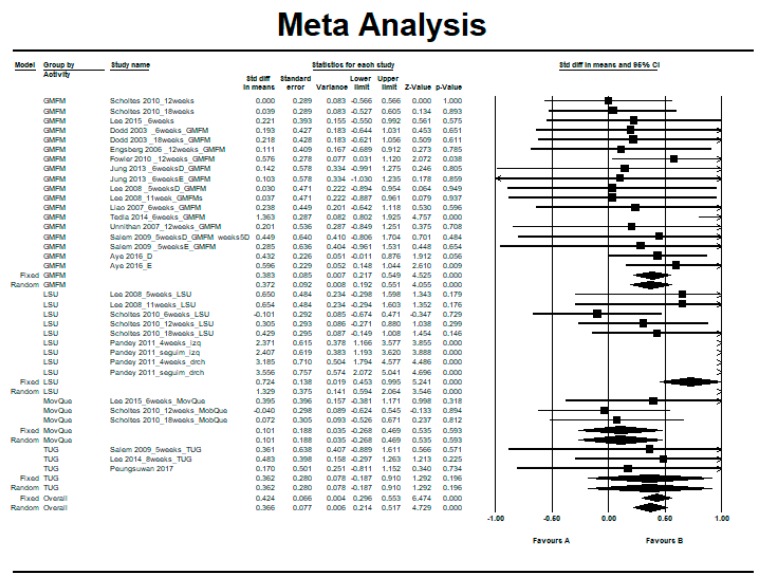
Pre–post intra-group difference in the intervention group with resistance therapy: all measurements and follow-ups.

Figure 2 shows the standardized mean difference (SMD) in a basic subgroup analysis according to the scale used in the study.

The individual results of the studies presented a moderate heterogeneity between them (Q = 79.64, degrees of freedom (df) = 32, *p* < 0.001, *I^2^* = 59.82%, Tau = 0.46) (see Table 4). The overall effect was in favor of the intervention, reaching statistical significance both in the fixed-effects model (SMD = 0.42, 95% CI = 0.30 to 0.55, *p* < 0.001) and in the random-effects model (SMD = 0.37, 95% CI = 0.21 to 0.52, *p* < 0.001) (see Figure 2).

Determinations were also restricted to the longer and shorter follow-ups for each article. No large differences were observed, with statistically significant SMDs of 0.38 (95% CI = 0.20 to 0.55) by restricting the determinations to the shorter follow-up, and 0.41 (95% CI = 0.24 to 0.59) by restricting the determinations to the longer follow-up under the random-effects model (data not shown in figures or tables).

Attending to the duration of the complete therapy, results showed a larger effect with an SMD of 0.75 (95% CI = 0.41 to 1.08), when the duration of the complete therapy was less than or equal to six weeks, and a smaller effect with an SMD of 0.18 (95% CI = −0.002 to 0.37), in studies with a longer duration of the complete therapy (from 7–12 weeks) (see Figure S1 and Table S1, Supplementary Materials).

Regarding the number of sessions, the results showed a difference in favor of studies with sessions applied fewer than three days a week with an SMD of 1.59 (95% CI = 0.67 to 2.50), compared to studies with sessions applied three days a week with an SMD of 0.32 (95% CI = 0.19 to 0.46) (see Figure S2 and Table S2, Supplementary Materials).

In relation to the duration of each session, studies showed a larger effect when the duration of the session was less than 30 min with an SMD of 1.08 (95% CI = 0.52 to 1.64) compared to studies that used between 30 and 60 min for each session with an SMD of 0.20 (95% CI = 0.03 to 0.38) or those that used between 60 and 90 min with an SMD of 0.57 (95% CI = −0.13 to 1.28) (see Figure S3 and Table S3, Supplementary Materials).

Regarding the protocol used, in seven studies, a “progressive strength training” protocol was used [9,13,14,33,34,37,39]. In three of the studies, the protocol used was a “table of resistance exercises” [10,12,38]. In four of the studies, the protocol used for resistance therapy was “functional training” [8,32,35,36], and, in one study, the protocol used for resistance therapy was a static bicycle [31]. The subgroup analysis showed a larger effect when using the “functional exercises” protocol with an SMD of 1.25 (95% CI = 0.46 to 2.04) (see Figure S4 and Table S4, Supplementary Materials).

Attending the methodological quality, the effect was in favor of intervention both in studies with an “excellent” score (SMD = 0.30, 95% CI = −0.29 to 0.88) or a “good” overall score (SMD = 0.55, 95% CI = 0.24 to 0.85) and in studies with a “fair” score (SMD = 0.47, 95% CI = −0.15 to 1.10) or “poor” score (SMD = 0.51, 95% CI = 0.22 to 0.80) (see Figure S5 and Table S5, Supplementary Materials).

### 3.2. Intra-Group Difference in the Control Group

**Figure 3:**
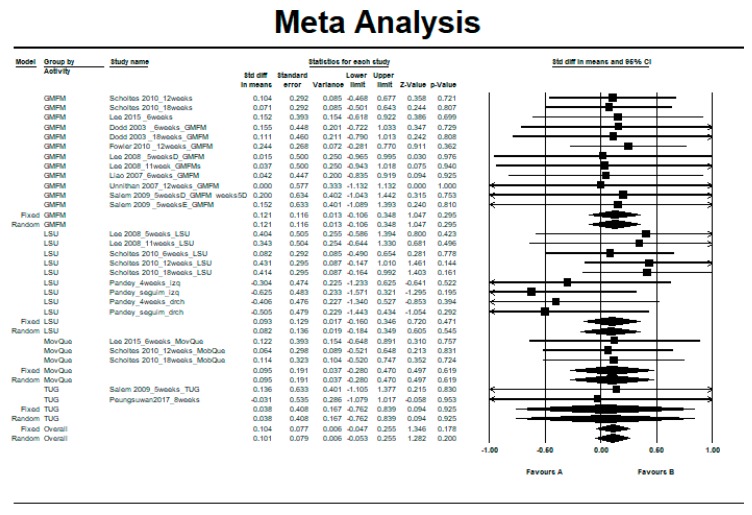
Pre–post intra-group difference in the control group.

Figure 3 shows the SMD in the control group, relative to the 10 controlled clinical trials [8,10,12,13,14,31,32,34,35,36].

The studies showed no heterogeneity between them (Q = 9.23, df = 25, *p* = 0.998, *I^2^* = 0%, Tau= 0) (see Table 5). The overall effect was in favor of the intervention, although only slightly and without reaching statistical significance using the fixed-effects model (SMD = 0.10, 95% CI = −0.05 to 0.26, *p* = 0.178) (see Figure 3). 

Figure S6 and Table S6 (Supplementary Materials) present a subgroup analysis according to the therapy used in the control group. When no therapy was used for the control group, the intra-group difference was against with an SMD of −0.15. In contrast, studies in which the control group received therapy were in favor with an overall SMD of 0.16.

### 3.3. Between-Group Difference

Eight studies [8,10,12,13,14,31,32,33,34] provided data related to a resistance therapy intervention that could be meta-analyzed for the evaluation of the between-group difference, i.e., between the intervention and control group, using the “GMFM” scale.

**Figure 4:**
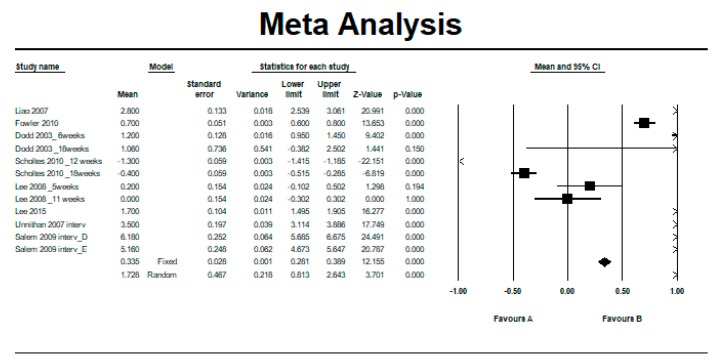
Pre–post difference of the Gross Motor Function Measure (GMFM) scale between groups in all follow-ups.

Heterogeneity was high (Q = 2720.45, df = 11, *p* = 0, *I^2^* = 99.60%, Tau = 1.60). The overall effect was in favor of intervention, reaching statistical significance using the fixed-effects model (MD = 0.34, 95% CI = 0.28 to 0.39, *p* < 0.001) and the random-effects model (MD = 1.73, 95% CI = 0.81 to 2.64, *p* < 0.001) (see Figure 4 and Table 6). 

### 3.4. Publication Bias

**Figure 5:**
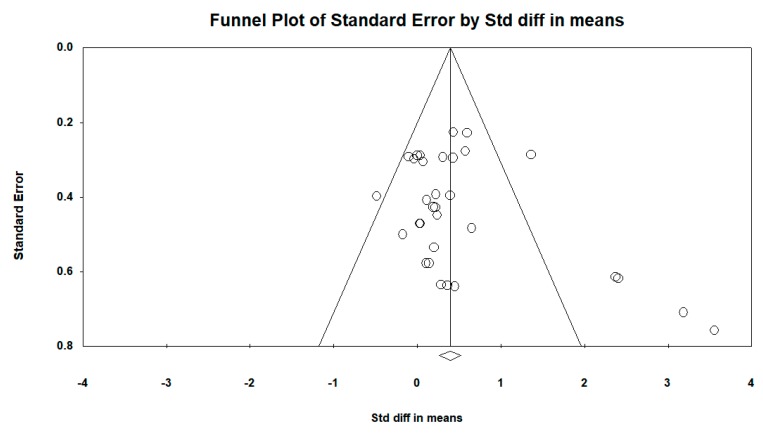
Funnel plot diagram of the standard error (*y*-axis) and the standardized mean difference (SMD) (*x*-axis): intra-group pre–post difference in the intervention group with resistance therapy in all follow-ups.

**Figure 6:**
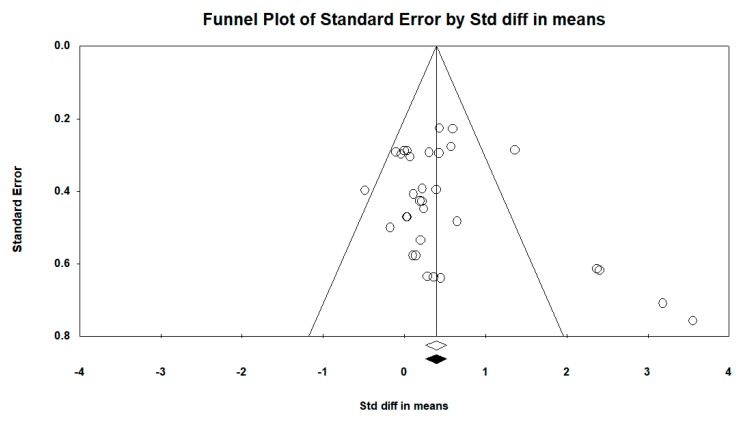
Funnel plot diagram of the standard error (*y*-axis) and the SMD (*x*-axis), after incorporating the Duval and Tweedie “trim and fill” procedure: pre–post intra-group difference in the intervention group with resistance therapy in all follow-ups.

In terms of publication bias in relation to the intra-group difference, the funnel plot was barely asymmetric. When incorporating the Duval and Tweedie “trim and fill” procedure, the model did not include any study; therefore, the overall effect adjusted by this procedure was similar to that observed (see Figure 5 and Figure 6).

**Figure 7:**
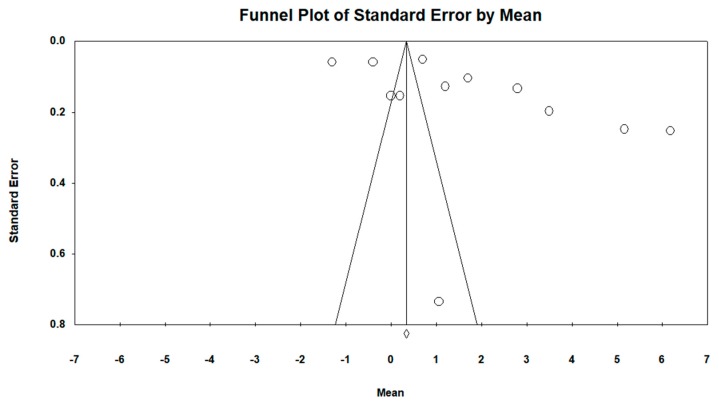
Funnel plot diagram of the standard error (*y*-axis) and the SMD (*x*-axis): difference between groups in the intervention group with resistance therapy on the GMFM scale in all follow-ups.

**Figure 8:**
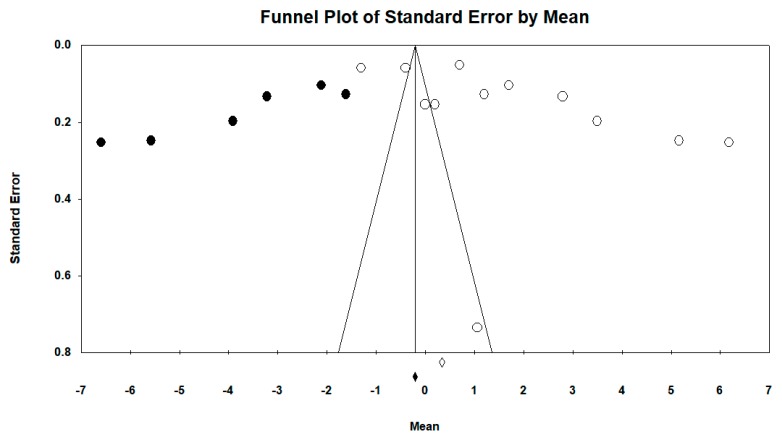
Funnel plot diagram of the standard error (*y*-axis) and the SMD (*x*-axis), after incorporating the Duval and Tweedie “trim and fill” procedure: difference between groups in the intervention group with resistance therapy on the GMFM scale in all follow-ups.

Regarding the difference in GMFM scale between groups, the funnel plot visually presented an asymmetry with a larger number of studies to the right (in favor of resistance therapy). When incorporating the Duval and Tweedie “trim and fill” procedure, the model included six studies from the left (represented as black circles). The best adjusted (unbiased) estimate using this procedure was, therefore, against intervention with an adjusted overall MD of −0.21 and −0.13 using the fixed-effects and random-effects models, respectively (see Figure 7 and Figure 8).

## 4. Discussion

In relation to the intra-group pre–post difference in motor function in the group intervened with resistance therapy, according to the criteria of Cohen et al. (1988) [40], the overall effect of the intervention would be small with an SMD of 0.37 under the random-effects model. The results support the absence of a publication bias in terms of intra-group results [26,27]. The result would be more favorable than that shown by Ryan et al. in their review published in 2017 [7], which reported a non-significant SMD of 0.12 (95% CI = −0.19 to 0.43), based on seven primary studies that included a total of 164 children and adolescents. 

Three out of the 15 studies were non-controlled one-arm clinical trials. By restricting the study to the 12 controlled clinical trials (with two arms), the overall effect size of the intervention was superior, with an SMD of 0.50 (random effects).

One study stood out for its positive impact on the LSU scale in terms of results and heterogeneity [35]. This study provided determinations at four weeks and at a follow-up of unspecified duration. In this study, the control group did not receive any intervention. This may be the explanation for these favorable results, since there was also another study where the control group did not receive any therapy [31], and the results stood out positively as well.

Another study [33] stood out for its positive result and its impact on heterogeneity on the GMFM scale. This is the only study in which children with greater motor impairment (levels I–V GMFCS (Gross Motor Function Classification System)) were considered among its inclusion criteria. This would explain its different results, more in favor than the rest of the studies.

Fifteen studies reported results on more than one scale. The results more favorable to the intervention were related to the LSU scale. The three studies that used this scale provided up to nine determinations on this scale, with an SMD of 1.33 [12,13,35]. It must be taken into account that the study with the most favorable results for the intervention [35], with an SMD of 3.56 in the longest follow-up, contributed with up to four determinations to the meta-analysis. It, therefore, had a greater positive influence on the overall effect.

Twelve of the 15 studies used the GMFM scale [8,9,10,12,13,14,31,32,33,34,37,39], making it the most used scale. Our results showed a statistically significant effect size of 0.37 in this scale, similar to the SMD of 0.38 reported in the review by Ryan et al. in 2017 [7].

The TUG scale was used in three out of the 15 studies [32,36,38], showing an SMD of 0.36.

The MobQue scale was the scale for which the effect size was the lowest (SMD = 0.10). Nevertheless, it was only used in two studies [13,14].

The GMFM scale and MobQue questionnaire show the score as a percentage, both measuring the motor function through a series of functional activities. The GMFM uses five dimensions, and the MobQue is a questionnaire of daily life activities of children with CP that is completed by their parents. The LSU and TUG tests evaluate more concrete activities; the former measures the full number of repetitions (upward and downward foot full movement) in which the child can participate on a step, and the latter measures the time it takes a person to get out of a chair, walk three meters, turn around, return to the chair, and sit.

With respect to the control group, the intra-group increase in motor function was lower (SMD = 0.10) (fixed effects) and did not reach statistical significance. There was no heterogeneity between the results in the studies. In eight of the 10 controlled studies, the control group received conventional therapy based on neurodevelopmental therapy, which included muscle stretching, functional exercises, and re-education of movement and gait. In two studies [31,35], no therapy was used for the control group. In these two studies, the intragroup difference was against with an SMD of −0.15, compared to the studies in which the control group received therapy, which showed an overall SMD in favor of 0.16.

Regarding the scales used, comparing the intra-group pre–post difference in the control group with the result discussed above in relation to the intervention group, we found the greatest differences in favor of the intervention group for the LSU scale, because the SMD was only 0.08 in the control group for this scale, followed by the GMFM scale (SMD = 0.12 in the control group) and the TUG scale (SMD = 0.04 in the control group). With respect to the MobQue scale, the results were similar in both groups (with similar SMDs of 0.10 in both control and intervention groups).

In relation to subgroup analysis based on the predefined sources of heterogeneity, the lower number of studies when forming the subgroups highlighted the individual influence of studies with more favorable results, such as the study by Pandey et al. [35], which used a 60-min “functional exercises” protocol, two days per week, with determinations at four weeks.

In any case, this analysis supports that a duration of six weeks or even less is enough to show a positive effect of the intervention, and that a longer duration (between seven and 12 weeks) does not appear to have a larger impact. Regarding the number of sessions, the difference between two and three sessions per week does not seem to have much clinical impact, if we disregard the influence of the study cited above [35]. In relation to the duration of each session, the results showed a smaller effect for longer training sessions (longer than 30 min); thus, fatigue would have to be taken into account. In this sense, a duration of each session of less than 30 min would lead to less fatigue, which seems to be associated with a larger effect of the intervention. The subgroup analysis shows a greater effect when using the “functional exercises” protocol, influenced once again by the favorable results of the study published by Pandey et al. (2011) [35].

These results do not coincide with the recommendations of the National Strength Training and Fitness Association (NSCA), which recommends, in healthy children, a training including 5–10 min of exercises using 50–85% of maximum resistance, 2–4 times per week for periods of 8–20 weeks [41]. None of the studies followed all NSCA recommendations. However, the response to training in children with CP is presumably quite different from the healthy population, and the adaptability of neural factors after resistance training may be reduced in these children [42]. Our results are similar to the results of the meta-analysis by Park et al., published in 2014 [6], which recommended interventions of 6–8 weeks of duration, with a frequency of 2–3 days per week; however, in our case, a slightly shorter duration (less than 30 min would be sufficient) is recommended rather than the 40–50 min in their meta-analysis. One of the limitations of resistance therapy is that long training programs can lead to demotivation at an early age, resulting in abandonment. It is, therefore, necessary to review and modify the games and exercises used to perform the resistance therapy, so as to guarantee adherence.

Depending on the methodological quality, the effect was in favor of intervention both in studies rated as “excellent” (*n* = 2) [32,36] or “good” (*n* = 7) [8,12,13,14,31,34,35] and in studies rated as “fair” (*n* = 6) [9,10,33,39] or “poor” (*n* = 2) [37,38], with no group scoring above an SMD of 0.5 (cut-off point considered for a moderate effect according to Cohen criteria).

Regarding the between-group difference in relation to the comparison of the GMFM scale, the mean differences (MDs) and their corresponding 95% CIs were shown under the random-effects model due to the high heterogeneity of the results (I^2^ > 99%). The possibility of obtaining the effect size on the natural scale allowed the results to be interpreted in non-standardized units of measurement.

In terms of the between-group difference, results restricted to the GMFM scale would support a positive difference in favor of resistance therapy with a mean difference of 1.73 points out of 100 (%). That is, despite the fact that children with cerebral palsy always achieve an increase in their motor function (determined through the GMFM scale), on average, the group intervened with resistance therapy obtained an increase of 1.73 points out of 100 on the GMFM scale with respect to the control group. This result is slightly lower than the two-point increase over 100 (95% CI = 0% to 4%) based on four studies (*n* = 99) [8,10,43,44] obtained in the meta-analysis published by Scianni et al., in 2009 [5].

The marked asymmetry of the funnel plot in the analysis supports the existence of a publication bias regarding the between-group difference, with controlled studies with more favorable results published more frequently. In this context, if we take into account the six studies from the left included when incorporating the Duval and Tweedle “trim and fill” procedure, the best adjusted (unbiased) estimate would actually be against intervention. This supports the need to register all clinical trials and the publication of all research protocols, along with the awareness-raising work of journal editors to ensure publication of results, either in favor or against the hypothesis. New studies must also consider the clinical relevance of the findings, from the points of view of both superiority and non-inferiority. In addition to the possibility of publication bias, another limitation of the present meta-analysis would be the low quality of some primary studies, and the small sample size of most of them.

## 5. Conclusions

In conclusion, our meta-analysis showed a statistically significant positive effect on motor function in favor of the use of resistance therapy in weakened musculature in children with CP. Resistance therapy would not only increase the strength of the musculature of children with CP, but this increased strength would also have an impact on the motor function. Nevertheless, it is possible that the between-group effect may have been overestimated due to the existence of a publication bias. All future randomized controlled clinical trials should be registered and published to address this issue. Future studies should also analyze the clinical relevance of the results, examining the most optimal regimens in terms of duration of therapy, number of sessions, duration of each session, and type of intervention protocol used.

## Figures and Tables

**Table 1:** Inclusion and exclusion criteria.

**Inclusion Criteria**
School-aged patients diagnosed with CP ^a^ (≤18 years) Design: both controlled (randomized or quasi-randomized) and non-controlled clinical trials Intervention based on resistance therapy Language: written in English or Spanish Information on at least one determination in motor function, both pre and post intervention, or differences between groups in these outcome measures in the case of controlled studies
**Exclusion Criteria**
Adult patients (≥19 years) Population with diseases other than CP Electrical stimulation as a resistance therapy of choice

^a^ Cerebral palsy.

**Table 2:** Characteristics of included studies.

First Author. Publication Year	Country	Study Design	Study Population	Duration	Procedure	Main Results	Other Results
Aye et al., 2016 [37]	Myanmar	Non-randomized controlled trial	40 CP ^a^ children (I–II GMFCS ^b^) Average age: 6 years	6 weeks 3 times/week 45 min/session	Progressive strength training	GMFM ^c^ D (*p* = 0.056): Pre: 28.4 ± 11.1 Post: 33.2 ± 11.1 GMFM E (*p* = 0.009): Pre: 42.4 ± 19.3 Post: 54.9 ± 22.5	
Dodd et al., 2003 [8]	Australia	Randomized controlled trial	21 CP children (I–III GMFCS) Average age: 13 years	6 weeks 3 times/week 20–30 min/session	Intervention group (*n* = 11): Functional strength training/conventional therapy Control group (*n* = 10): Conventional therapy	GMFM: intervention group (*p* = 0.651): Pre: 64.2 ± 27.8 Post: 69 ± 21.4 Control group (*p* = 0.729): Pre: 71.7 ± 24.9 Post: 75.3 ± 21.3	Gait speed (cm/s): Intervention group (*p* = 0.950): Pre: 79 ± 38.83 Post: 80 ± 35.33 Control group: Pre: 82.5 ± 40.83 Post: 84.16 ± 34.66
Engsberg et al., 2006 [9]	USA	Randomized controlled trial	12 CP children (I–III GMFCS) Average age: 9.9 years	12 weeks 3 times/week	Intervention group (*n* = 9): Progressive strength training. Control group (*n* = 3): Non-therapy	GMFM: Intervention group (*p* = 0.785): Pre: 65.8 ± 30.8 Post: 69.1 ± 28.4	Gait speed (cm/s): Intervention group (*p* = 0.743): Pre: 85.9 ± 31.1 Post: 91 ± 34.6 Control group: Pre: 80.1 ± 23.4 Post: 78.6 ± 31.3 Cadence (steps/min): Intervention group (*p* = 0.813): Pre: 120.3 ± 36.3 Post: 124.4 ± 37.2 Control group: Pre: 121.7 ± 17.9 Post: 123.1 ± 12.9 Step length (cm): Intervention group (*p* = 0.826): Pre: 82.6 ± 21 Post: 84.8 ± 21.4 Control group: Pre: 80.6 ± 14.8 Post: 77.7 ± 25.8
Fowler et al., 2010 [31]	USA	Randomized controlled trial	62 CP children (I–III GMFCS) Age: 7–18 years	12 weeks 3 times/week 60 min/session	Intervention group (*n* = 31): Resistance training on bicycle Control group (*n* = 31): Non-therapy	GMFM: Intervention group (*p* = 0.038): Pre: 69.6 ± 2.1 Post: 70.8 ± 2.07 Control group (*p* = 0.382): Pre: 68.8 ± 2.12 Post: 69.3 ± 1.97	Gait speed (cm/s): Intervention group (*p* = 0.282): Pre: 111.5 ± 6.67 Post: 113.33 ± 6.2 Control group: Pre: 97.83 ± 6.05 Post: 103.5 ± 6.26
Jung et al., 2013 [39]	South Korea	Non-randomized controlled trial	6 CP children (I GMFCS) Age: 4–10 years	6 weeks 3 times/week 30 min/session	Progressive strength training	GMFM D (*p* = 0.805): Pre: 88.5 ± 15 Post: 90.6 ± 14.5 GMFM E (*p* = 0.859): Pre: 78 ± 21.9 Post: 80.3 ± 22.9	Gait speed (cm/s) (*p* = 0.512): Pre: 81.4 ± 19 Post: 88.7 ± 19.2 Cadence (steps/min) (*p* = 0.039): Pre: 117.7 ± 10.7 Post: 129.6 ± 7.1 Step length (cm) (*p* = 0.607): Pre: 84 ± 15.9 Post: 88.9 ± 16.9
Lee et al., 2008 [12]	Korea	Randomized controlled trial	17 CP children (II–III GMFCS) Age: 4–12 years	5 weeks 3 times/week 60 min/session	Intervention group (*n* = 9): Table of resistance exercises Control group (*n* = 8): Conventional neurodevelopmental therapy	GMFM: Intervention group (*p* = 0.949): Pre: 86.5 ± 13.3 Post: 86.9 ± 13.4 Control group (*p* = 0.976): Pre: 85.2 ± 13.4 Post: 85.4 ± 13.5	Gait speed (cm/s): Intervention group (*p* = 0.236): Pre: 54.7 ± 30.7 Post: 74.6 ± 38.7 Control group: Pre: 69.8 ± 43 Post: 68.2 ± 42.9 Cadence (steps/min): Intervention group (*p* = 0.848): Pre: 106.8 ± 37.1 Post: 109.7 ± 26 Control group: Pre: 107.9 ± 48.4 Post: 101.1 ± 47.4 Step length (cm): Intervention group (*p* = 0.137): Pre: 62.5 ± 21.8 Post: 80 ± 26.4 Control group: Pre: 70 ± 32.1 Post: 68.2 ± 42.9
Lee et al., 2014 [38]	Korea	Non-randomized controlled trial	13 CP children (I–II GMFCS) Age: 6–18 years	8 weeks 2 times/week 30 min/session	Table of resistance exercises	TUG ^d^ (*p* = 0.225): Pre: 25.17 ± 7.53 Post: 21.79 ± 6.43	
Lee et al., 2015 [14]	Korea	Randomized controlled trial	26 CP children (I–III GMFCS) Age: 5–10 years	6 weeks 3 times/week 50 min/session	Intervention group (*n* = 13): Functional strength training/conventional neurodevelopmental therapy Control group (*n* = 13): Conventional neurodevelopmental therapy	GMFM: Intervention group (*p* = 0.575): Pre: 78 ± 19.1 Post: 81.9 ± 16.1 Control group (*p* = 0.699): Pre: 79.1 ± 14.7 Post: 81.3 ± 14.3 MobQue ^e^: Intervention group (*p* = 0.318): Pre: 55.7 ± 29.9 Post: 66.9 ± 26.7 Control group (*p* = 0.757): Pre: 48.3 ± 26.9 Post: 51.5 ± 25.7	
Liao et al., 2007 [10]	China	Randomized controlled trial	20 CP children (I–II GMFCS) Age: 5–12 years	6 weeks 3 times/week 90 min/session	Intervention group (*n* = 10): Table of resistance exercises/conventional neurodevelopmental therapy Control group (*n* = 10): Conventional neurodevelopmental therapy	GMFM: Intervention group (*p* = 0.596): Pre: 76.6 ± 13.91 Post: 79.8 ± 12.96 Control group (*p* = 0.925): Pre: 83.1 ± 10.11 Post: 83.5 ± 8.85	
Pandey et al., 2011 [35]	India	Randomized controlled trial	18 CP children Age: 5–10 years	4 weeks 2 times/week 60 min/session	Intervention group (*n* = 9): Functional strength training Control group (*n* = 9): Non-therapy	Lateral step up (left): Intervention group (*p* < 0.001): Pre: 6.22 ± 1.44 Post: 11.8 ± 3 Control group (*p* = 0.522): Pre: 6.44 ± 1.66 Post: 6 ± 1.2 Lateral step up (right): Intervention group (*p* = 0.000): Pre: 6.4 ± 1.6 Post: 12.6 ± 2.24 Control group (*p* = 0.394): Pre: 5.8 ± 1.2 Post: 5.34 ± 1.06	Gait speed: Intervention group (*p* = 0.001): Pre: 54 ± 8 Post: 70 ± 10 Control group: Pre: 59 ± 9 Post: 60 ± 10 Cadence: Intervention group (*p* = 0.004): Pre: 111 ± 10 Post: 127 ± 11 Control group: Pre: 125 ± 27 Post: 127 ± 26 Step length: Intervention group (*p* = 1.000): Pre: 63 ± 16 Post: 63 ± 10 Control group: Pre: 58 ± 14 Post: 60 ± 10
Peungsuwan et al., 2017 [36]	Thailand	Randomized controlled trial	15 CP children (I–III GMFCS) Age: 7–16 years	8 weeks 3 times/week 70 min/session	Intervention group (*n* = 8): Functional strength training/conventional therapy Control group (*n* = 7): Conventional therapy	TUG: Intervention group (*p* = 0.734): Pre: 10.1 ± 3.1 Post: 9.5 ± 3.9 Control group (*p* = 0.953): Pre: 11.6 ± 3 Post: 11.7 ± 3.4	Gait speed (cm/s): Intervention group (*p* = 0.280): Pre: 100 ± 20 Post: 111 ± 20 Control group: Pre: 111 ± 20 Post: 99 ± 20
Salem et al., 2009 [32]	USA	Randomized controlled trial	10 CP children (I–III GMFCS) Average age: 8 years	5 weeks 2 times/week	Intervention group (*n* = 5): Functional strength training Control group (*n* = 5): Conventional therapy	GMFM D: Intervention group (*p* = 0.484): Pre: 62.02 ± 25.24 Post: 73.3 ± 25.04 Control group (*p* = 0.753): Pre: 64.6 ± 26.07 Post: 69.7 ± 24.97 GMFM E: Intervention group (*p* = 0.654): Pre: 45.62 ± 31.57 Post: 54.66 ± 31.9 Control group (*p* = 0.810): Pre: 49.42 ± 25.42 Post: 53.3 ± 25.62 TUG: Intervention group (*p* = 0.571): Pre: 19.8 ± 11.32 Post: 15.8 ± 10.83 Control group (*p* = 0.830): Pre: 23.6 ± 13.09 Post: 11.7 ± 3.4	
Scholtes et al., 2010 [13]	The Netherlands	Randomized controlled trial	51 CP children (I–III GMFCS) Age:6–13 years	12 weeks 3 times/week 60 min/session	Intervention group (*n* = 26): Functional strength training Control group (*n* = 25): Conventional therapy	GMFM: Intervention group (*p* = 1.000): Pre: 76.1 ± 12.8 Post: 76.1 ± 11.8 Control group (*p* = 0.721): Pre: 71.8 ± 12.5 Post: 73.1 ± 12.4 Lateral step up: Intervention group (*p* = 0.299): Pre: 15.6 ± 4 Post: 17 ± 5.1 Control group (*p* = 0.144): Pre: 13.3 ± 5.4 Post: 15.4 ± 4.3 MobQue: Intervention group (*p* = 0.894): Pre: 68.42 ± 20.93 Post: 67.51 ± 24.5 Control group (*p* = 0.831): Pre: 64.77 ± 26.26 Post: 66.4 ± 25.93	
Tedla et al., 2014 [33]	Saudi Arabia	Randomized controlled trial	60 CP children (I–V GMFCS) Age: 5–14 years	6 weeks 3 times/week 60–90 min/session	Intervention group (*n* = 30): Progressive strength training. Control group (*n* = 30): Conventional therapy	GMFM (*p* < 0.001): Intervention group: Pre: 69.02 ± 7.64 Post: 78.84 ± 6.45	
Unnithan et al., 2007 [34]	United Kingdom and Greece	Randomized controlled trial	13 CP children (II–III GMFCS) Age:14–18 years	12 weeks 3 times/week 70 min/session	Intervention group (*n* = 7): Progressive strength training/conventional neurodevelopmental therapy Control group (*n* = 6): Conventional neurodevelopmental therapy	GMFM: Intervention group (*p* = 0.708): Pre: 30.35 ± 16.95 Post: 33.85 ± 17.87 Control group (*p* = 1.000): Pre: 30.76 ± 12.52 Post: 30.76 ± 12.52	

^a^ Cerebral palsy; ^b^ Gross Motor Function Classification System; ^c^ Gross Motor Function Measure; ^d^ Time Up and Go; ^e^ Mobility Questionnaire. USA—United States of America.

**Table 3:** Results of the quality assessment based on the Physiotherapy Evidence Database (PEDro) Scale, for the studies finally included.

Criteria	Aye	Dodd	Engsberg	Fowler	Jung	Lee	Lee	Lee	Pandey	Peungsuwan	Liao	Salem	Scholtes	Tedla	Unnithan
	2016	2003	2006	2010	2013	2008	2014	2015	2011	2017	2007	2009	2010	2014	2007
Eligibility criteria were specified ^a^	1	1	1	1	0	1	1	0	1	1	1	0	1	1	1
Random allocation	n.a. ^b^	1	1	1	n.a.	1	n.a.	1	1	1	1	1	1	1	1
Cancelled allocation	n.a.	1	0	0	n.a.	1	n.a.	0	1	1	0	1	1	0	0
Groups similar at baseline	n.a.	0	0	1	n.a.	0	n.a.	0	0	1	0	0	0	0	1
Subject blinding	n.a.	0	0	0	n.a.	0	n.a.	0	0	0	0	0	0	0	0
Therapist blinding	n.a.	0	0	0	n.a.	0	n.a.	0	0	0	0	0	0	0	0
Assessor blinding	n.a.	1	0	1	n.a.	0	n.a.	1	1	1	1	1	1	0	0
<15% dropout	0	1	0	1	1	1	0	1	1	1	0	1	1	1	1
Intention-to-treat analysis	n.a.	0	0	0	n.a.	1	n.a.	1	0	1	0	1	0	0	1
Between-group statistical comparisons	1	1	1	1	1	1	1	1	1	1	1	1	1	1	1
Point measures and variability data	1	1	1	1	1	1	1	1	1	1	1	1	1	1	1
Total score (0/10)	2	6	3	6	3	6	2	6	6	8	4	7	6	4	6

^a^ Criterium that does not contribute to the total score because it evaluates the external validity of the study; ^b^ n.a. = not applicable (non-controlled studies, only intervention group).

**Table 4:** Global heterogeneity in the intervention group: all scales and follow-ups.

Scales	*N* of Determinations	Heterogeneity					
		Q	df	*p* (χ^2^)	*I^2^* (%)	τ^2^	τ
All scales and all follow-ups	33	79.64	32	0.000	59.82	0.21	0.46
GMFM ^A^	18	19.00	17	0.328	10.54	0.02	0.13
LSU ^B^	9	51.64	8	0.000	84.51	1.00	1.00
MobQue ^C^	3	0.78	2	0.676	0.00	0.00	0.00
TUG ^D^	3	0.24	2	0.888	0.00	0.00	0.00

^A^ Gross Motor Function Measure; ^B^ Lateral Step Up; ^C^ Mobility Questionnaire; ^D^ Time Up and Go. df—degrees of freedom.

**Table 5:** Global heterogeneity in the control group: all scales and follow-ups.

Scales	*N* of Determinations	Heterogeneity					
		Q	df	*p* (χ^2^)	*I^2^* (%)	τ^2^	τ
All scales and all follow-ups	26	9.23	25	0.998	0.00	0.00	0.00
GMFM ^A^	12	0.42	11	1.000	0.00	0.00	0.00
LSU ^B^	9	8.69	8	0.369	7.97	0.01	0.12
MobQue ^C^	3	0.02	2	0.990	0.00	0.00	0.00
TUG ^D^	2	0.04	1	0.840	0.00	0.00	0.00

^A^ Gross Motor Function Measure; ^B^ Lateral Step Up; ^C^ Mobility Questionnaire; ^D^ Time Up and Go.

**Table 6:** Global heterogeneity between groups on the GMFM scale: all follow-ups.

Difference between Groups Using GMFM ^A^	*N* of Determinations	Heterogeneity					
		Q	df	*P* (χ^2^)	*I^2^* (%)	τ^2^	τ
All follow-ups	12	2720.45	11	0	99.60	2.56	1.60

^A^ Gross Motor Function Measure.

## Supplementary Materials

The following are available online at https://www.mdpi.com/1660-4601/16/22/4513/s1: Figure S1: Pre–post intra-group difference in the intervention group with resistance therapy. Analysis of subgroups according to the complete duration of the therapy. All measurements and follow-ups; Figure S2: Pre–post intra-group difference in the intervention group with resistance therapy. Analysis of subgroups according to the number of sessions. All measurements and follow-ups; Figure S3: Pre–post intra-group difference in the intervention group with resistance therapy. Analysis of subgroups according to the duration of the session. All measurements and follow-ups; Figure S4: Pre–post intra-group difference in the intervention group with resistance therapy. Analysis of subgroups according to the intervention protocol. All measurements and follow-ups; Figure S5: Pre–post intra-group difference in the intervention group with resistance therapy. Analysis of subgroups according to the methodological quality. All measurements and follow-ups; Figure S6: Pre–post intra-group difference in the control group. Analysis of subgroups according to the therapy used. All measurements and follow-ups; Table S1: Global heterogeneity in the intervention group with resistance therapy, according to the complete duration of the therapy. All measurements and follow-ups; Table S2: Global heterogeneity in the intervention group with resistance therapy, according to the number of sessions. All measurements and follow-ups; Table S3: Global heterogeneity in the intervention group with resistance therapy, according to the duration of the session. All measurements and follow-ups; Table S4: Global heterogeneity in the intervention group with resistance therapy, according to the intervention protocol. All measurements and follow-ups; Table S5: Global heterogeneity in the intervention group with resistance therapy. Analysis of subgroups, according to the methodological quality All measurements and follow-ups; Table S6: Global heterogeneity in the control group. Analysis of subgroups, according to the therapy used. All measurements and follow-ups; Checklist S1. PRISMA.
