# Supplementary material for: Impact of Resistance Therapy on Motor Function in Children with Cerebral Palsy: A Systematic Review and Meta-Analysis
Source: Int J Environ Res Public Health. 2019 Nov 15;16(22):4513. doi: 10.3390/ijerph16224513 (PMC6888121; doi:10.3390/ijerph16224513)
Supplement: Supplementary file 1 [file ijerph-16-04513-s001.pdf]

International Journal of
Environmental Research and Public Health

MDPI

Supplementary Materials: Impact of Resistance Therapy on Motor Function in Children with Cerebral Palsy: A Systematic Review and Meta-Analysis

Luisa Collado-Garrido [1], Paula Parás-Bravo [2,3,*], Pilar Calvo-Martín [1] and Miguel Santibáñez-Margüello [2,3,4]

1 Rehabilitation Service, The Marqués de Valdecilla University Hospital, Valdecilla Avenue s/n. C.P.: 39008 Santander, Cantabria, Spain; luisacolladogarrido@yahoo.es (L.C.-G.); mpilar.calvo@scsalud.es (P.C.-M.)
2 Faculty of Nursing, University of Cantabria, Valdecilla Avenue s/n., 39008 Santander, Cantabria, Spain; paula.paras@unican.es (P.P.-B.); miguel.santibanez@unican.es (M. S.-M.).
3 Research Nursing Group IDIVAL; Cardenal Herrera Oria Street s/n. C.P., 3901 Cantabria, Spain; paula.paras@unican.es (P.P.-B.).
4 Global Health Research Group, University of Cantabria, 39008 Santander, Cantabria, Spain; miguel.santibanez@unican.es (M. S.-M.)
* Correspondence: paula.paras@unican.es

Table S1. Global heterogeneity in the intervened group with resisted therapy, according to the complete duration of the therapy. All measurements and follow-ups.

Intervention group Duration of the therapy	N determinations	Heterogeneity					
		Q	df	p (Chi^2)	I^2 (%)	τ^2	τ
All measurements and follow-ups	33	79.636	32	0.000	59.817	0.214	0.462
<6 weeks	21	60.868	20	0.000	67.142	0.373	0.611
7–12 weeks	12	5.753	11	0.889	0.000	0.000	0.000

Table S2. Global heterogeneity in the intervened group with resisted therapy, according to the number of sessions. All measurements and follow-ups.

Intervention group Number of sessions	N determinations	Heterogeneity					
		Q	df	p (Chi^2)	I^2 (%)	τ^2	τ
All measurements and follow-ups	32	79.636	31	0.000	59.817	0.214	0.462
<3 sessionss	8	32.783	7	0.000	78.648	1.343	1.159
3 sessions	25	24.912	24	0.411	3.660	0.005	0.068

Table S3. Global heterogeneity in the group intervened with resisted therapy, according to the duration of the session. All measurements and follow-ups.

Intervention group Duration of session	N determinations	Heterogeneity					
		Q	df	p (Chi^2)	I^2 (%)	τ^2	τ
All measurements and follow-ups	29	78.966	28	0.000	64.542	0.244	0.494

< 30 minutes	11	48.016	10	0.000	79.174	0.651	0.807
30- 60 minutes	14	7.474	13	0.876	0.000	0.000	0.000
60 -90 minutes	4	8.251	3	0.041	63.642	0.319	0.565

Table S4. Global heterogeneity in the intervened group with resisted therapy, according to the intervention protocol. All measurements and follow-ups.

Intervention group Intervention protocol	N determinations	Heterogeneity					
		Q	df	p (Chi^2)	I^2 (%)	τ^2	τ
All measurements and follow-ups	33	79.636	32	0.000	59.817	0.214	0.462
Progressive Strength Training	16	22.118	15	0.105	32.183	0.048	0.220
Table of Resisted Exercises	6	1.854	5	0.869	0.000	0.000	0.000
Functional training	10	43.652	9	0.000	79.382	1.255	1.120
Static bicycle	1	0.000	0	1.000	0.000	0.000	0.000

Table S5. Global heterogeneity in the group intervened with resisted therapy. Analysis of subgroups, according to the methodological quality All measurements and follow-ups.

Intervention group Methodological quality	N determinations	Heterogeneity					
		Q	df	p (Chi^2)	I^2 (%)	τ^2	τ
All measurements and follow-ups	33	79.636	32	0.000	59.817	0.214	0.462
Excelent	4	0.131	3	0.988	0.000	0.000	0.000
Godd	21	66.109	20	0.000	69.747	0.333	0.577
Fair	5	10.443	4	0.034	61.697	0.302	0.550
Po	3	0.265	2	0.876	0.000	0.000	0.000

Table S6. Global heterogeneity in the control group. Analysis of subgroups, according to the therapy used. All measurements and follow-ups.

Control group	N determinations	Heterogeneity					
		Q	df	p (Chi^2)	I^2 (%)	τ^2	τ
All studies	26	9.23	25	0.998	0.00	0.00	0.00
Conventional therapy used in the control group	21	2.07	20	1.000	0.00	0.00	0.00
No therapy used in the control group	5	4.07	4	0.396	1.77	0.00	0.06

Meta Analysis

Model	Group by duration	Study name	Std diff in means	Standard error	Variance	Lower limit	Upper limit	Z-Value	p-Value
	<6 weeks	Lee 2015 _6weeks	0.221	0.393	0.155	-0.550	0.992	0.561	0.575
	<6 weeks	Dodd 2003 _6weeks_GMFM	0.193	0.427	0.183	-0.644	1.031	0.453	0.651
	<6 weeks	Dodd 2003 _18weeks_GMFM	0.218	0.428	0.183	-0.621	1.056	0.509	0.611
	<6 weeks	Jung 2013 _6weeksD_GMFM	0.142	0.578	0.334	-0.991	1.275	0.246	0.805
	<6 weeks	Jung 2013 _6weeksE_GMFM	0.103	0.578	0.334	-1.030	1.235	0.178	0.859
	<6 weeks	Lee 2008 _5weeksD_GMFM	0.030	0.471	0.222	-0.894	0.954	0.064	0.949
	<6 weeks	Lee 2008_11week_GMFMs	0.037	0.471	0.222	-0.887	0.961	0.079	0.937
	<6 weeks	Liao 2007_6weeks_GMFM	0.238	0.449	0.201	-0.642	1.118	0.530	0.596
	<6 weeks	Tedla 2014_6weeks_GMFM	1.363	0.287	0.082	0.802	1.925	4.757	0.000
	<6 weeks	Salem 2009_5weeksD_GMFM weeks5D	0.449	0.640	0.410	-0.806	1.704	0.701	0.484
	<6 weeks	Salem 2009 _5weeksE_GMFM	0.285	0.636	0.404	-0.961	1.531	0.448	0.654
	<6 weeks	Aye 2016_D	0.432	0.226	0.051	-0.011	0.876	1.912	0.056
	<6 weeks	Aye 2016_E	0.596	0.229	0.052	0.148	1.044	2.610	0.009
	<6 weeks	Lee 2015_6weeks_MovQue	0.395	0.396	0.157	-0.381	1.171	0.998	0.318
	<6 weeks	Lee 2008_5weeks_LSU	0.650	0.484	0.234	-0.298	1.598	1.343	0.179
	<6 weeks	Lee 2008_11weeks_LSU	0.654	0.484	0.234	-0.294	1.603	1.352	0.176
	<6 weeks	Pandey 2011_4weeks_left	2.371	0.615	0.378	1.166	3.577	3.855	0.000
	<6 weeks	Pandey 2011_follow_left	2.407	0.619	0.383	1.193	3.620	3.888	0.000
	<6 weeks	Pandey 2011_4weeks_right	3.185	0.710	0.504	1.794	4.577	4.486	0.000
	<6 weeks	Pandey 2011_follow_right	3.556	0.757	0.574	2.072	5.041	4.696	0.000
	<6 weeks	Salem 2009_5weeks_TUG	0.361	0.638	0.407	-0.889	1.611	0.566	0.571
Fixed	<6 weeks		0.654	0.091	0.008	0.475	0.834	7.154	0.000
Random	<6 weeks		0.745	0.171	0.029	0.410	1.080	4.354	0.000
	7-12 weeks	Scholtes 2010_12weeks	0.000	0.289	0.083	-0.566	0.566	0.000	1.000
	7-12 weeks	Scholtes 2010_18weeks	0.039	0.289	0.083	-0.527	0.605	0.134	0.893
	7-12 weeks	Engsberg 2006 _12weeks_GMFM	0.111	0.409	0.167	-0.689	0.912	0.273	0.785
	7-12 weeks	Fowler 2010 _12weeks_GMFM	0.576	0.278	0.077	0.031	1.120	2.072	0.038
	7-12 weeks	Unnithan 2007_12weeks_GMFM	0.201	0.536	0.287	-0.849	1.251	0.375	0.708
	7-12 weeks	Scholtes 2010_12weeks_MobQue	-0.040	0.298	0.089	-0.624	0.545	-0.133	0.894
	7-12 weeks	Scholtes 2010_18weeks_MobQue	0.072	0.305	0.093	-0.526	0.671	0.237	0.812
	7-12 weeks	Scholtes 2010_6weeks_LSU	-0.101	0.292	0.085	-0.674	0.471	-0.347	0.729
	7-12 weeks	Scholtes 2010_12weeks_LSU	0.305	0.293	0.086	-0.271	0.880	1.038	0.299
	7-12 weeks	Scholtes 2010_18weeks_LSU	0.429	0.295	0.087	-0.149	1.008	1.454	0.146
	7-12 weeks	Lee 2014_8weeks_TUG	0.483	0.398	0.158	-0.297	1.263	1.213	0.225
	7-12 weeks	Peungsuwan 2017	0.170	0.501	0.251	-0.811	1.152	0.340	0.734
Fixed	7-12 weeks		0.182	0.094	0.009	-0.002	0.366	1.936	0.053
Random	7-12 weeks		0.182	0.094	0.009	-0.002	0.366	1.936	0.053
Fixed	Overall		0.424	0.066	0.004	0.296	0.553	6.474	0.000
Random	Overall		0.312	0.082	0.007	0.151	0.473	3.792	0.000

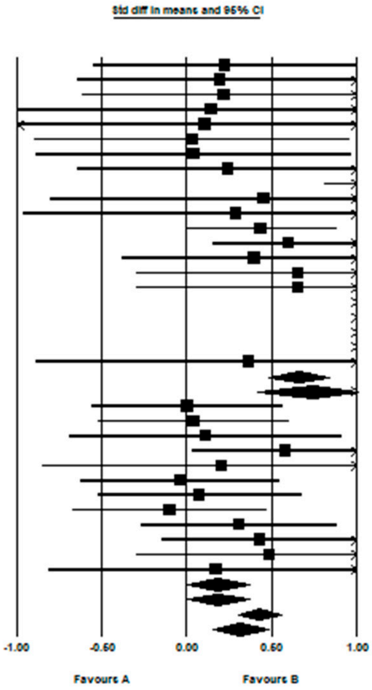

Meta Analysis

Figure S1. Pre-post intra-group difference in the intervened group with resistance therapy. Analysis of subgroups according to the complete duration of the therapy. All measurements and follow-ups.

Meta Analysis

Model	Group by sessions	Study name	Std diff in means	Standard error	Variance	Lower limit	Upper limit	Z-Value	p-Value
	< 3 sessions	Salem 2009_5weeksD_GMFM weeks5D	0.449	0.640	0.410	-0.806	1.704	0.701	0.484
	< 3 sessions	Salem 2009 _5weeksE_GMFM	0.285	0.636	0.404	-0.961	1.531	0.448	0.654
	< 3 sessions	Pandey 2011_4weeks_left	2.371	0.615	0.378	1.166	3.577	3.855	0.000
	< 3 sessions	Pandey 2011_follow_left	2.407	0.619	0.383	1.193	3.620	3.888	0.000
	< 3 sessions	Pandey 2011_4weeks_right	3.185	0.710	0.504	1.794	4.577	4.486	0.000
	< 3 sessions	Pandey 2011_follow_right	3.556	0.757	0.574	2.072	5.041	4.696	0.000
	< 3 sessions	Salem 2009_5weeks_TUG	0.361	0.638	0.407	-0.889	1.611	0.566	0.571
	< 3 sessions	Lee 2014_8weeks_TUG	0.483	0.398	0.158	-0.297	1.263	1.213	0.225
Fixed	< 3 sessions		1.359	0.210	0.044	0.947	1.770	6.470	0.000
Random	< 3 sessions		1.585	0.466	0.217	0.672	2.499	3.401	0.001
	3 sessions	Scholtes 2010_12weeks	0.000	0.289	0.083	-0.566	0.566	0.000	1.000
	3 sessions	Scholtes 2010_18weeks	0.039	0.289	0.083	-0.527	0.605	0.134	0.893
	3 sessions	Lee 2015 _6weeks	0.221	0.393	0.155	-0.550	0.992	0.561	0.575
	3 sessions	Dodd 2003 _6weeks_GMFM	0.193	0.427	0.183	-0.644	1.031	0.453	0.651
	3 sessions	Dodd 2003 _18weeks_GMFM	0.218	0.428	0.183	-0.621	1.056	0.509	0.611
	3 sessions	Engsberg 2006 _12weeks_GMFM	0.111	0.409	0.167	-0.689	0.912	0.273	0.785
	3 sessions	Fowler 2010 _12weeks_GMFM	0.576	0.278	0.077	0.031	1.120	2.072	0.038
	3 sessions	Jung 2013 _6weeksD_GMFM	0.142	0.578	0.334	-0.991	1.275	0.246	0.805
	3 sessions	Jung 2013 _6weeksE_GMFM	0.103	0.578	0.334	-1.030	1.235	0.178	0.859
	3 sessions	Lee 2008 _5weeksD_GMFM	0.030	0.471	0.222	-0.894	0.954	0.064	0.949
	3 sessions	Lee 2008_11week_GMFMs	0.037	0.471	0.222	-0.887	0.961	0.079	0.937
	3 sessions	Liao 2007_6weeks_GMFM	0.238	0.449	0.201	-0.642	1.118	0.530	0.596
	3 sessions	Tedla 2014_6weeks_GMFM	1.363	0.287	0.082	0.802	1.925	4.757	0.000
	3 sessions	Unnithan 2007_12weeks_GMFM	0.201	0.536	0.287	-0.849	1.251	0.375	0.708
	3 sessions	Aye 2016_D	0.432	0.226	0.051	-0.011	0.876	1.912	0.056
	3 sessions	Aye 2016_E	0.596	0.229	0.052	0.148	1.044	2.610	0.009
	3 sessions	Lee 2015_6weeks_MovQue	0.395	0.396	0.157	-0.381	1.171	0.998	0.318
	3 sessions	Scholtes 2010_12weeks_MobQue	-0.040	0.298	0.089	-0.624	0.545	-0.133	0.894
	3 sessions	Scholtes 2010_18weeks_MobQue	0.072	0.305	0.093	-0.526	0.671	0.237	0.812
	3 sessions	Lee 2008_5weeks_LSU	0.650	0.484	0.234	-0.298	1.598	1.343	0.179
	3 sessions	Lee 2008_11weeks_LSU	0.654	0.484	0.234	-0.294	1.603	1.352	0.176
	3 sessions	Scholtes 2010_6weeks_LSU	-0.101	0.292	0.085	-0.674	0.471	-0.347	0.729
	3 sessions	Scholtes 2010_12weeks_LSU	0.305	0.293	0.086	-0.271	0.880	1.038	0.299
	3 sessions	Scholtes 2010_18weeks_LSU	0.429	0.295	0.087	-0.149	1.008	1.454	0.146
	3 sessions	Peungsuwan 2017	0.170	0.501	0.251	-0.811	1.152	0.340	0.734
Fixed	3 sessions		0.323	0.069	0.005	0.188	0.459	4.690	0.000
Random	3 sessions		0.321	0.071	0.005	0.183	0.460	4.547	0.000
Fixed	Overall		0.424	0.066	0.004	0.296	0.553	6.474	0.000
Random	Overall		0.350	0.070	0.005	0.213	0.487	5.006	0.000

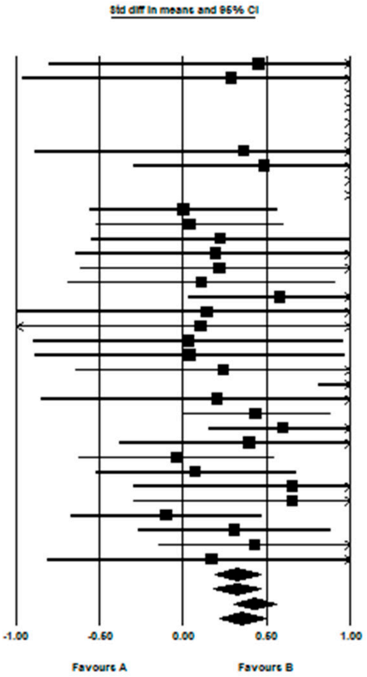

Meta Analysis

Figure S2. Pre-post intra-group difference in the intervened group with resistance therapy. Analysis of subgroups according to the number of sessions. All measurements and follow-ups.

Meta Analysis

Model	Group by duration	Study name	Std diff in means	Standard error	Variance	Lower limit	Upper limit	Z-Value	p-Value
	<6 weeks	Lee 2015 _6weeks	0.221	0.393	0.155	-0.550	0.992	0.561	0.575
	<6 weeks	Dodd 2003 _6weeks_GMFM	0.193	0.427	0.183	-0.644	1.031	0.453	0.651
	<6 weeks	Dodd 2003 _18weeks_GMFM	0.218	0.428	0.183	-0.621	1.056	0.509	0.611
	<6 weeks	Jung 2013 _6weeksD_GMFM	0.142	0.578	0.334	-0.991	1.275	0.246	0.805
	<6 weeks	Jung 2013 _6weeksE_GMFM	0.103	0.578	0.334	-1.030	1.235	0.178	0.859
	<6 weeks	Lee 2008 _5weeksD_GMFM	0.030	0.471	0.222	-0.894	0.954	0.064	0.949
	<6 weeks	Lee 2008_11week_GMFMs	0.037	0.471	0.222	-0.887	0.961	0.079	0.937
	<6 weeks	Liao 2007_6weeks_GMFM	0.238	0.449	0.201	-0.642	1.118	0.530	0.596
	<6 weeks	Tedla 2014_6weeks_GMFM	1.363	0.287	0.082	0.802	1.925	4.757	0.000
	<6 weeks	Salem 2009_5weeksD_GMFM weeks5D	0.449	0.640	0.410	-0.806	1.704	0.701	0.484
	<6 weeks	Salem 2009 _5weeksE_GMFM	0.285	0.636	0.404	-0.961	1.531	0.448	0.654
	<6 weeks	Aye 2016_D	0.432	0.226	0.051	-0.011	0.876	1.912	0.056
	<6 weeks	Aye 2016_E	0.596	0.229	0.052	0.148	1.044	2.610	0.009
	<6 weeks	Lee 2015_6weeks_MovQue	0.395	0.396	0.157	-0.381	1.171	0.998	0.318
	<6 weeks	Lee 2008_5weeks_LSU	0.650	0.484	0.234	-0.298	1.598	1.343	0.179
	<6 weeks	Lee 2008_11weeks_LSU	0.654	0.484	0.234	-0.294	1.603	1.352	0.176
	<6 weeks	Pandey 2011_4weeks_left	2.371	0.615	0.378	1.166	3.577	3.855	0.000
	<6 weeks	Pandey 2011_follow_left	2.407	0.619	0.383	1.193	3.620	3.888	0.000
	<6 weeks	Pandey 2011_4weeks_right	3.185	0.710	0.504	1.794	4.577	4.486	0.000
	<6 weeks	Pandey 2011_follow_right	3.556	0.757	0.574	2.072	5.041	4.696	0.000
	<6 weeks	Salem 2009_5weeks_TUG	0.361	0.638	0.407	-0.889	1.611	0.566	0.571
Fixed	<6 weeks		0.654	0.091	0.008	0.475	0.834	7.154	0.000
Random	<6 weeks		0.745	0.171	0.029	0.410	1.080	4.354	0.000
	7-12 weeks	Scholtes 2010_12weeks	0.000	0.289	0.083	-0.566	0.566	0.000	1.000
	7-12 weeks	Scholtes 2010_18weeks	0.039	0.289	0.083	-0.527	0.605	0.134	0.893
	7-12 weeks	Engsberg 2006 _12weeks_GMFM	0.111	0.409	0.167	-0.689	0.912	0.273	0.785
	7-12 weeks	Fowler 2010 _12weeks_GMFM	0.576	0.278	0.077	0.031	1.120	2.072	0.038
	7-12 weeks	Unnithan 2007_12weeks_GMFM	0.201	0.536	0.287	-0.849	1.251	0.375	0.708
	7-12 weeks	Scholtes 2010_12weeks_MobQue	-0.040	0.298	0.089	-0.624	0.545	-0.133	0.894
	7-12 weeks	Scholtes 2010_18weeks_MobQue	0.072	0.305	0.093	-0.526	0.671	0.237	0.812
	7-12 weeks	Scholtes 2010_6weeks_LSU	-0.101	0.292	0.085	-0.674	0.471	-0.347	0.729
	7-12 weeks	Scholtes 2010_12weeks_LSU	0.305	0.293	0.086	-0.271	0.880	1.038	0.299
	7-12 weeks	Scholtes 2010_18weeks_LSU	0.429	0.295	0.087	-0.149	1.008	1.454	0.146
	7-12 weeks	Lee 2014_8weeks_TUG	0.483	0.398	0.158	-0.297	1.263	1.213	0.225
	7-12 weeks	Peungsuwan 2017	0.170	0.501	0.251	-0.811	1.152	0.340	0.734
Fixed	7-12 weeks		0.182	0.094	0.009	-0.002	0.366	1.936	0.053
Random	7-12 weeks		0.182	0.094	0.009	-0.002	0.366	1.936	0.053
Fixed	Overall		0.424	0.066	0.004	0.296	0.553	6.474	0.000
Random	Overall		0.312	0.082	0.007	0.151	0.473	3.792	0.000

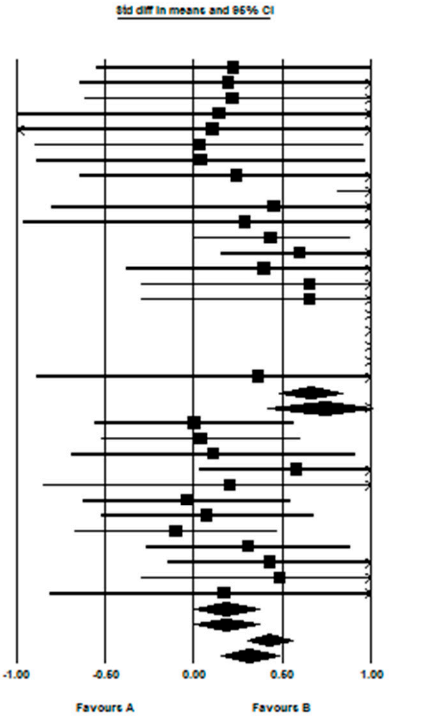

Meta Analysis

Figure S3. Pre-post intra-group difference in the intervened group with resistance therapy. Analysis of subgroups according to the duration of the session. All measurements and follow-ups.

Meta Analysis

Model	Group by Protocol	Study name	Std diff in means	Standard error	Variance	Lower limit	Upper limit	Z-Value	p-Value
	Bicycle	Fowler 2010 _12weeks_GMFM	0.576	0.278	0.077	0.031	1.120	2.072	0.038
Fixed	Bicycle		0.576	0.278	0.077	0.031	1.120	2.072	0.038
Random	Bicycle		0.576	0.278	0.077	0.031	1.120	2.072	0.038
	Functional Training	Dodd 2003 _6weeks_GMFM	0.193	0.427	0.183	-0.644	1.031	0.453	0.651
	Functional Training	Dodd 2003 _18weeks_GMFM	0.218	0.428	0.183	-0.621	1.056	0.509	0.611
	Functional Training	Salem 2009_5weeksD_GMFM weeks5D	0.449	0.640	0.410	-0.806	1.704	0.701	0.484
	Functional Training	Salem 2009 _5weeksE_GMFM	0.285	0.636	0.404	-0.961	1.531	0.448	0.654
	Functional Training	Pandey 2011_4weeks_left	2.371	0.615	0.378	1.166	3.577	3.855	0.000
	Functional Training	Pandey 2011_follow_left	2.407	0.619	0.383	1.193	3.620	3.888	0.000
	Functional Training	Pandey 2011_4weeks_right	3.185	0.710	0.504	1.794	4.577	4.486	0.000
	Functional Training	Pandey 2011_follow_right	3.556	0.757	0.574	2.072	5.041	4.696	0.000
	Functional Training	Salem 2009_5weeks_TUG	0.361	0.638	0.407	-0.889	1.611	0.566	0.571
	Functional Training	Peungsuwan 2017	0.170	0.501	0.251	-0.811	1.152	0.340	0.734
Fixed	Functional Training		0.981	0.179	0.032	0.630	1.331	5.486	0.000
Random	Functional Training		1.248	0.402	0.161	0.461	2.035	3.107	0.002
	PST	Scholtes 2010_12weeks	0.000	0.289	0.083	-0.566	0.566	0.000	1.000
	PST	Scholtes 2010_18weeks	0.039	0.289	0.083	-0.527	0.605	0.134	0.893
	PST	Lee 2015 _6weeks	0.221	0.393	0.155	-0.550	0.992	0.561	0.575
	PST	Engsberg 2006 _12weeks_GMFM	0.111	0.409	0.167	-0.689	0.912	0.273	0.785
	PST	Jung 2013 _6weeksD_GMFM	0.142	0.578	0.334	-0.991	1.275	0.246	0.805
	PST	Jung 2013 _6weeksE_GMFM	0.103	0.578	0.334	-1.030	1.235	0.178	0.859
	PST	Tedla 2014_6weeks_GMFM	1.363	0.287	0.082	0.802	1.925	4.757	0.000
	PST	Unnithan 2007_12weeks_GMFM	0.201	0.536	0.287	-0.849	1.251	0.375	0.708
	PST	Aye 2016_D	0.432	0.226	0.051	-0.011	0.876	1.912	0.056
	PST	Aye 2016_E	0.596	0.229	0.052	0.148	1.044	2.610	0.009
	PST	Lee 2015_6weeks_MovQue	0.395	0.396	0.157	-0.381	1.171	0.998	0.318
	PST	Scholtes 2010_12weeks_MobQue	-0.040	0.298	0.089	-0.624	0.545	-0.133	0.894
	PST	Scholtes 2010_18weeks_MobQue	0.072	0.305	0.093	-0.526	0.671	0.237	0.812
	PST	Scholtes 2010_6weeks_LSU	-0.101	0.292	0.085	-0.674	0.471	-0.347	0.729
	PST	Scholtes 2010_12weeks_LSU	0.305	0.293	0.086	-0.271	0.880	1.038	0.299
	PST	Scholtes 2010_18weeks_LSU	0.429	0.295	0.087	-0.149	1.008	1.454	0.146
Fixed	PST		0.316	0.079	0.006	0.161	0.471	4.000	0.000
Random	PST		0.300	0.099	0.010	0.107	0.494	3.038	0.002
	Resisted Training	Lee 2008 _5weeksD_GMFM	0.030	0.471	0.222	-0.894	0.954	0.064	0.949
	Resisted Training	Lee 2008_11week_GMFMs	0.037	0.471	0.222	-0.887	0.961	0.079	0.937
	Resisted Training	Liao 2007_6weeks_GMFM	0.238	0.449	0.201	-0.642	1.118	0.530	0.596
	Resisted Training	Lee 2008_5weeks_LSU	0.650	0.484	0.234	-0.298	1.598	1.343	0.179
	Resisted Training	Lee 2008_11weeks_LSU	0.654	0.484	0.234	-0.294	1.603	1.352	0.176
	Resisted Training	Lee 2014_8weeks_TUG	0.483	0.398	0.158	-0.297	1.263	1.213	0.225
Fixed	Resisted Training		0.351	0.186	0.035	-0.014	0.716	1.882	0.060
Random	Resisted Training		0.351	0.186	0.035	-0.014	0.716	1.882	0.060
Fixed	Overall		0.424	0.066	0.004	0.296	0.553	6.474	0.000
Random	Overall		0.373	0.082	0.007	0.213	0.533	4.570	0.000

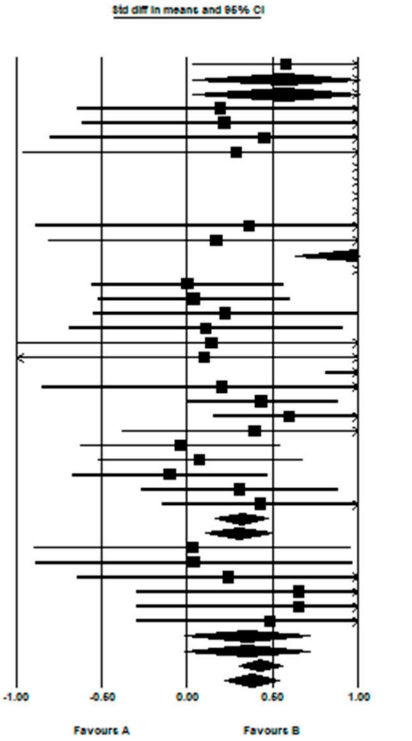

Meta Analysis

Figure S4. Pre-post intra-group difference in the intervened group with resistance therapy. Analysis of subgroups according to the the intervention protocol. All measurements and follow-ups.

Meta Analysis

Model	Group by Quality	Study name	Std diff in means	Standard error	Variance	Lower limit	Upper limit	Z-Value	p-Value
	Excelent	Salem 2009_5weeksD_GMFM weeks5D	0.449	0.640	0.410	-0.806	1.704	0.701	0.484
	Excelent	Salem 2009 _5weeksE_GMFM	0.285	0.636	0.404	-0.961	1.531	0.448	0.654
	Excelent	Salem 2009_5weeks_TUG	0.361	0.638	0.407	-0.889	1.611	0.566	0.571
	Excelent	Peungsuwan 2017	0.170	0.501	0.251	-0.811	1.152	0.340	0.734
Fixed	Excelent		0.296	0.297	0.088	-0.285	0.878	0.999	0.318
Random	Excelent		0.296	0.297	0.088	-0.285	0.878	0.999	0.318
	Fair	Engsberg 2006 _12weeks_GMFM	0.111	0.409	0.167	-0.689	0.912	0.273	0.785
	Fair	Jung 2013 _6weeksD_GMFM	0.142	0.578	0.334	-0.991	1.275	0.246	0.805
	Fair	Jung 2013 _6weeksE_GMFM	0.103	0.578	0.334	-1.030	1.235	0.178	0.859
	Fair	Liao 2007_6weeks_GMFM	0.238	0.449	0.201	-0.642	1.118	0.530	0.596
	Fair	Tedla 2014_6weeks_GMFM	1.363	0.287	0.082	0.802	1.925	4.757	0.000
Fixed	Fair		0.659	0.185	0.034	0.295	1.022	3.554	0.000
Random	Fair		0.471	0.318	0.101	-0.153	1.095	1.479	0.139
	Good	Scholtes 2010_12weeks	0.000	0.289	0.083	-0.566	0.566	0.000	1.000
	Good	Scholtes 2010_18weeks	0.039	0.289	0.083	-0.527	0.605	0.134	0.893
	Good	Lee 2015 _6weeks	0.221	0.393	0.155	-0.550	0.992	0.561	0.575
	Good	Dodd 2003 _6weeks_GMFM	0.193	0.427	0.183	-0.644	1.031	0.453	0.651
	Good	Dodd 2003 _18weeks_GMFM	0.218	0.428	0.183	-0.621	1.056	0.509	0.611
	Good	Fowler 2010 _12weeks_GMFM	0.576	0.278	0.077	0.031	1.120	2.072	0.038
	Good	Lee 2008 _5weeksD_GMFM	0.030	0.471	0.222	-0.894	0.954	0.064	0.949
	Good	Lee 2008_11week_GMFMs	0.037	0.471	0.222	-0.887	0.961	0.079	0.937
	Good	Unnithan 2007_12weeks_GMFM	0.201	0.536	0.287	-0.849	1.251	0.375	0.708
	Good	Lee 2015_6weeks_MovQue	0.395	0.396	0.157	-0.381	1.171	0.998	0.318
	Good	Scholtes 2010_12weeks_MobQue	-0.040	0.298	0.089	-0.624	0.545	-0.133	0.894
	Good	Scholtes 2010_18weeks_MobQue	0.072	0.305	0.093	-0.526	0.671	0.237	0.812
	Good	Lee 2008_5weeks_LSU	0.650	0.484	0.234	-0.298	1.598	1.343	0.179
	Good	Lee 2008_11weeks_LSU	0.654	0.484	0.234	-0.294	1.603	1.352	0.176
	Good	Scholtes 2010_6weeks_LSU	-0.101	0.292	0.085	-0.674	0.471	-0.347	0.729
	Good	Scholtes 2010_12weeks_LSU	0.305	0.293	0.086	-0.271	0.880	1.038	0.299
	Good	Scholtes 2010_18weeks_LSU	0.429	0.295	0.087	-0.149	1.008	1.454	0.146
	Good	Pandey 2011_4weeks_left	2.371	0.615	0.378	1.166	3.577	3.855	0.000
	Good	Pandey 2011_follow_left	2.407	0.619	0.383	1.193	3.620	3.888	0.000
	Good	Pandey 2011_4weeks_right	3.185	0.710	0.504	1.794	4.577	4.486	0.000
	Good	Pandey 2011_follow_right	3.556	0.757	0.574	2.072	5.041	4.696	0.000
Fixed	Good		0.362	0.082	0.007	0.200	0.523	4.394	0.000
Random	Good		0.545	0.156	0.024	0.239	0.851	3.486	0.000
	Poor	Aye 2016_D	0.432	0.226	0.051	-0.011	0.876	1.912	0.056
	Poor	Aye 2016_E	0.596	0.229	0.052	0.148	1.044	2.610	0.009
	Poor	Lee 2014_8weeks_TUG	0.483	0.398	0.158	-0.297	1.263	1.213	0.225
Fixed	Poor		0.509	0.149	0.022	0.217	0.801	3.416	0.001
Random	Poor		0.509	0.149	0.022	0.217	0.801	3.416	0.001
Fixed	Overall		0.424	0.066	0.004	0.296	0.553	6.474	0.000
Random	Overall		0.497	0.097	0.009	0.307	0.686	5.142	0.000

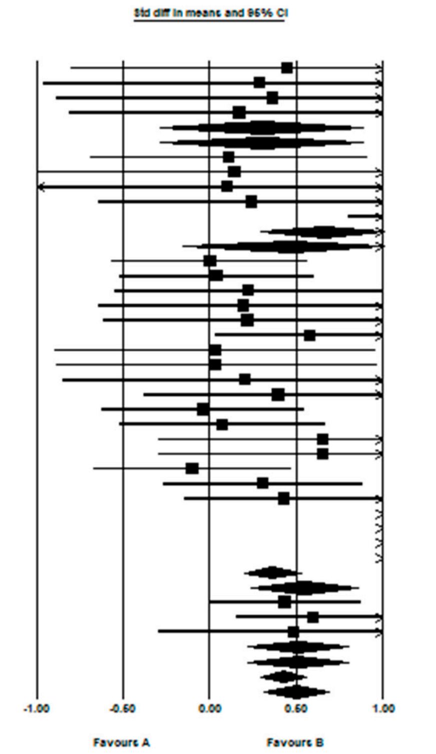

Meta Analysis

Figure S5. Pre-post intra-group difference in the intervened group with resistance therapy. Analysis of subgroups according to the methodological quality. All measurements and follow-ups.

Meta Analysis

Model	Group by Therapy	Study name	Std diff in means	Standard error	Variance	Lower limit	Upper limit	Z-Value	p-Value
	Conventional therapy	Scholtes 2010 12weeks	0.104	0.292	0.085	-0.468	0.677	0.358	0.721
	Conventional therapy	Scholtes 2010 18weeks	0.071	0.292	0.085	-0.501	0.643	0.244	0.807
	Conventional therapy	Lee 2015 6weeks	0.152	0.393	0.154	-0.618	0.922	0.386	0.699
	Conventional therapy	Dodd 2003 6weeks GMFM	0.155	0.448	0.201	-0.722	1.033	0.347	0.729
	Conventional therapy	Dodd 2003 18weeks GMFM	0.111	0.460	0.211	-0.790	1.013	0.242	0.808
	Conventional therapy	Lee 2008 5weeksD GMFM	0.015	0.500	0.250	-0.965	0.995	0.030	0.976
	Conventional therapy	Lee 2008 11week GMFMs	0.037	0.500	0.250	-0.943	1.018	0.075	0.940
	Conventional therapy	Liao 2007 6weeks GMFM	0.042	0.447	0.200	-0.835	0.919	0.094	0.925
	Conventional therapy	Unnithan 2007 12weeks GMFM	0.000	0.577	0.333	-1.132	1.132	0.000	1.000
	Conventional therapy	Salem 2009 5weeksD GMFM weeks5D	0.200	0.634	0.402	-1.043	1.442	0.315	0.753
	Conventional therapy	Salem 2009 5weeksE GMFM	0.152	0.633	0.401	-1.089	1.393	0.240	0.810
	Conventional therapy	Lee 2015 6weeks MovQue	0.122	0.393	0.154	-0.648	0.891	0.310	0.757
	Conventional therapy	Scholtes 2010 12weeks MobQue	0.064	0.298	0.089	-0.521	0.648	0.213	0.831
	Conventional therapy	Scholtes 2010 18weeks MobQue	0.114	0.323	0.104	-0.520	0.747	0.352	0.724
	Conventional therapy	Lee 2008 5weeks LSU	0.404	0.505	0.255	-0.586	1.394	0.800	0.423
	Conventional therapy	Lee 2008 11weeks LSU	0.343	0.504	0.254	-0.644	1.330	0.681	0.496
	Conventional therapy	Scholtes 2010 6weeks LSU	0.082	0.292	0.085	-0.490	0.654	0.281	0.778
	Conventional therapy	Scholtes 2010 12weeks LSU	0.431	0.295	0.087	-0.147	1.010	1.461	0.144
	Conventional therapy	Scholtes 2010 18weeks LSU	0.414	0.295	0.087	-0.164	0.992	1.403	0.161
	Conventional therapy	Salem 2009 5weeks TUG	0.136	0.633	0.401	-1.105	1.377	0.215	0.830
	Conventional therapy	Peungsuwan2017 8weeks	-0.031	0.535	0.286	-1.079	1.017	-0.058	0.953
Fixed	Conventional therapy		0.162	0.086	0.007	-0.006	0.329	1.889	0.059
Random	Conventional therapy		0.162	0.086	0.007	-0.006	0.329	1.889	0.059
	Non-therapy	Fowler 2010 12weeks GMFM	0.244	0.268	0.072	-0.281	0.770	0.911	0.362
	Non-therapy	Pandey 4weeks izq	-0.304	0.474	0.225	-1.233	0.625	-0.641	0.522
	Non-therapy	Pandey seguim izq	-0.625	0.483	0.233	-1.571	0.321	-1.295	0.195
	Non-therapy	Pandey 4weeks drch	-0.406	0.476	0.227	-1.340	0.527	-0.853	0.394
	Non-therapy	Pandey seguim drch	-0.505	0.479	0.229	-1.443	0.434	-1.054	0.292
Fixed	Non-therapy		-0.147	0.178	0.032	-0.497	0.202	-0.826	0.409
Random	Non-therapy		-0.153	0.181	0.033	-0.507	0.202	-0.843	0.399
Fixed	Overall		0.104	0.077	0.006	-0.047	0.255	1.346	0.178
Random	Overall		0.104	0.077	0.006	-0.047	0.256	1.347	0.178

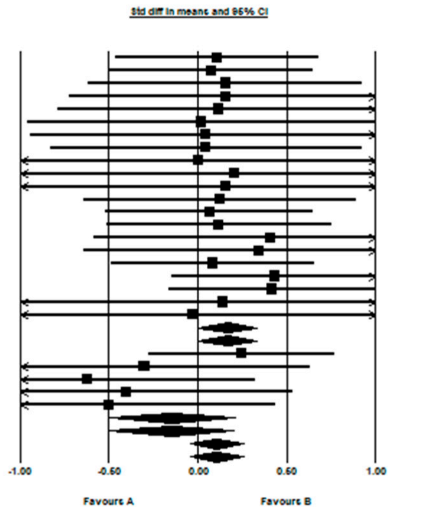

Meta Analysis

Figure S6. Pre-post intra-group difference in the control group. Analysis of subgroups according to the therapy used. All measurements and follow-ups.
